# Reactive oxygen species contribute to delirium-like behavior by activating CypA/MMP9 signaling and inducing blood-brain barrier impairment in aged mice following anesthesia and surgery

**DOI:** 10.3389/fnagi.2022.1021129

**Published:** 2022-10-19

**Authors:** Li-fang Liu, Yun Hu, Yi-nuo Liu, De-wen Shi, Chang Liu, Xin Da, Si-hui Zhu, Qian-yun Zhu, Ji-qian Zhang, Guang-hong Xu

**Affiliations:** ^1^Department of Anesthesiology, The First Affiliated Hospital of Anhui Medical University, Hefei, China; ^2^Key Laboratory of Anesthesia and Perioperative Medicine of Anhui Higher Education Institutes, Hefei, China

**Keywords:** reactive oxygen species, postoperative delirium, cyclophilin A, matrix metalloproteinase 9, blood-brain barrier

## Abstract

Postoperative delirium (POD) is common in the elderly and is associated with poor clinical outcomes. Reactive oxygen species (ROS) and blood-brain barrier (BBB) damage have been implicated in the development of POD, but the association between these two factors and the potential mechanism is not clear. Cyclophilin A (CypA) is a specifically chemotactic leukocyte factor that can be secreted in response to ROS, which activates matrix metalloproteinase 9 (MMP9) and mediates BBB breakdown. We, therefore, hypothesized that ROS may contribute to anesthesia/surgery-induced BBB damage and delirium-like behavior *via* the CypA/MMP9 pathway. To test these hypotheses, 16-month-old mice were subjected to laparotomy under 3% sevoflurane anesthesia (anesthesia/surgery) for 3 h. ROS scavenger (N-acetyl-cysteine) and CypA inhibitor (Cyclosporin A) were used 0.5 h before anesthesia/surgery. A battery of behavior tests (buried food test, open field test, and Y maze test) was employed to evaluate behavioral changes at 24 h before and after surgery in the mice. Levels of tight junction proteins, CypA, MMP9, postsynaptic density protein (PSD)-95, and synaptophysin in the prefrontal cortex were assessed by western blotting. The amounts of ROS and IgG in the cortex of mice were observed by fluorescent staining. The concentration of S100β in the serum was detected by ELISA. ROS scavenger prevented the reduction in TJ proteins and restored the permeability of BBB as well as reduced the levels of CypA/MMP9, and further alleviated delirium-like behavior induced by anesthesia/surgery. Furthermore, the CypA inhibitor abolished the increased levels of CypA/MMP, which reversed BBB damage and ameliorated delirium-like behavior caused by ROS accumulation. Our findings demonstrated that ROS may participate in regulating BBB permeability in aged mice with POD *via* the CypA/MMP9 pathway, suggesting that CypA may be a potential molecular target for preventing POD.

## Introduction

Postoperative delirium (POD) is an acute and fluctuating mental state change, characterized by fluctuating disturbances in attention, level of consciousness, and cognitive defects, which usually occur within a few days after surgery (Aldecoa et al., 2017; Evered et al., 2018). In the past, delirium was considered an acute and reversible brain dysfunction. However, studies have shown that delirium is related to accelerated cognitive decline and Alzheimer’s disease and related dementia (ADRD) (Witlox et al., 2010; Fong et al., 2015). POD can occur at any age, and the elderly are susceptible, which probably results from age-related neurovascular dysfunction and degenerative changes in the central nervous system (CNS) (Witlox et al., 2010; Inouye et al., 2014; Strom et al., 2014), especially neurons within the prefrontal cortex (PFC) are less resilient to stress (McEwen and Morrison, 2013). With the increase in aging of the global population, the demand of elderly people for anesthesia/surgery treatments has been growing, as has the incidence of POD. At present, the pathogenesis of POD has not been fully identified, so there is no standard knowledge and methods to reduce the occurrence of POD.

Although the pathogenesis of POD remains poorly defined, more data have shown that the abnormally activated oxidative stress and the breakdown of the blood-brain barrier (BBB) are positively correlated with the development of POD (Karlidag et al., 2006; Taylor et al., 2022). Oxidative stress plays a key role in the pathogenesis mechanism of nervous system diseases (Patel, 2016), by promoting Aβ deposition, tau hyperphosphorylation, and the subsequent synapses and neuron loss (Chen and Zhong, 2014; Anwar, 2022). Specifically, anesthesia/surgery result in excessive reactive oxygen species (ROS) production (Peng et al., 2016) and reduce the activities of some antioxidant enzymes such as catalase (CAT), superoxide dismutase (SOD), and glutathione peroxidase (GSH-Px) (Zhang et al., 2018), as well as inducing delirium-like behavior in mice, indicating that oxidative stress is of great importance to the development of POD. However, it is not clear how oxidative stress induces the occurrence of POD. The BBB affords a safe environment for the CNS, and its breakdown acts as an early biomarker of cognitive impairment, independent of Aβ or tau biomarker changes (Nation et al., 2019). BBB disruption is a consistent pathological feature of several neurodegenerative diseases associated with cognitive decline (Cai et al., 2018). Hughes et al. (2016) found that endothelial dysfunction and impaired microvascular permeability have also been observed in critical illness patients with delirium, by assessing plasma biomarkers such as S100 calcium-binding protein B (S100β), plasminogen activator inhibitor-1, and E-selectin. Moreover, Yang et al. (2017) confirmed that anesthesia and surgery can induce age-associated BBB dysfunction and cognitive impairment in mice, which was significantly related to the duration of anesthesia (Wen et al., 2020). Oxidative stress can disrupt tight junction (TJ) proteins and increase the permeability of BBB (Abdul-Muneer et al., 2013; Song et al., 2014), which in turn makes the brain more vulnerable to stress. Disruption of BBB strengthens the relationship between intraoperative oxidative damage and delirium as well as neuronal injury (Lopez et al., 2020). These studies suggest that the abnormal accumulation of ROS may be the underlying mechanism of POD, in which the impairment of BBB function is of vital importance. However, it remains uncertain whether ROS regulate BBB permeability in response to anesthesia/surgery and the potential mechanism needs further exploration.

Cyclophilin A (CypA) is a specifically directed chemotactic leukocyte factor with a strong pro-inflammatory effect that can activate matrix metalloproteinases (MMPs) (Garimella et al., 2020). The matrix metalloproteinases are zinc-dependent endopeptidases that damage the BBB by directly degrading extracellular matrix proteins and tight junction proteins, and the abnormal expression of matrix metalloproteinase 9 (MMP9) is directly related to the destruction of BBB (Alluri et al., 2016; Turner and Sharp, 2016). Previous studies have shown that the CypA/MMP9 signaling pathway is responsible for BBB breakdown and subsequent cognitive decline, thus the blockade of CypA/MMP9 signaling can restore BBB integrity and normalize the function of neurons and synapses (Bell et al., 2012; Halliday et al., 2016; Montagne et al., 2020). In pericytes, CypA can activate the CD147 receptor and downstream NF-kappa B, induce MMP9 expression for the degradation of BBB function (Pan et al., 2020). However, the mechanism by which anesthesia/surgery affects the expression of CypA has not been reported, and the interaction between ROS and CypA is not completely clear. Previous studies have shown that CypA can be secreted in response to ROS in various cells, and the amount of secretion increases with the degree of oxidative stress (Cao et al., 2019). Therefore, we assumed that the CypA/MMP9 pathway may be engaged in the development of delirium-like behavior induced by anesthesia/surgery, and that ROS-induced BBB disruption may be a possible pathway that should be explored.

The objective of the current study was to determine whether CypA contributed to anesthesia/surgery-induced BBB damage and delirium-like behavior, and whether ROS regulated CypA expression for these effects, which could provide further evidence for the mechanism and new insights into the prevention of POD. To test the hypothesis, *N*-acetyl-cysteine (NAC) was used to observe whether scavenging ROS could improve BBB destruction and alleviate delirium-like behavior in aged mice. NAC is an antioxidant and free-radical scavenging agent (Bavarsad Shahripour et al., 2014), which can consequently protect against oxidative stress and may have neuroprotective effects, as demonstrated by its efficacy in reducing markers of oxidative stress and the severity of cognitive dysfunction in animal models (Skvarc et al., 2017). To determine whether BBB disruption induced by ROS was through CypA/MMP9 pathway and could be corrected with cyclosporine A (CsA), a drug that binds and inhibits the effect of CypA (Handschumacher et al., 1984), we used a low dose of CsA previously shown not to cause systemic or central nervous system toxicity and effectively reversed CypA-driven BBB breakdown (Santos and Schauwecker, 2003; Bell et al., 2012; Nikolakopoulou et al., 2021).

## Materials and methods

### Animals

The Biomedical Ethics Committee of Anhui Medical University has approved our animal protocol. All experiments were performed in compliance with the National Institutes of Health Guide for the Care and Use of Laboratory Animals. Efforts were made to minimize the number of animals used. This study did not involve human subjects and was not pre-registered. C57BL/6J female mice (16 months old, weighing 30–40 g) were purchased from Skbex Biotechnology Company, Henan, China. All animals were group-housed at five per cage on a normal day/night cycle with free access to food and water. The temperature and humidity were maintained according to the standards established by the experimental animal laboratory. Before starting the experiment, the mice were allowed 1 week to acclimatize to the laboratory environment.

### Surgical model

We only used female mice without studying male mice in this experiment, as the latest research has reported that there is no difference in the effects of sex on the physiological state or behaviors in mice (Shansky, 2019). Mice were randomly divided into the following six groups: control group, anesthesia/surgery group, control plus NAC group, anesthesia/surgery plus NAC group, control plus CsA group, anesthesia/surgery plus CsA group, where NAC was used as a ROS scavenger and CsA was used as a CypA inhibitor. The mouse model of POD was established by laparotomy under 3% sevoflurane general anesthesia. The sevoflurane concentration of 3% is clinically relevant, and studies have shown that the administration of 3% sevoflurane does not significantly alter blood gases in mice (Satomoto et al., 2009). We, therefore, did not measure blood gases in the present study. Specifically, mice were placed in an anesthesia chamber with 3% sevoflurane plus 60% oxygen (balanced with nitrogen). An anesthetic gas monitor was used to measure the concentration of sevoflurane in real time. Fifteen minutes after the induction, the mouse was taken out of the chamber and placed on a heating thermostat to maintain the body temperature between 36 and 37°C during the surgery. Sevoflurane anesthesia was maintained with a nose mask, and 1% lidocaine was used to assist analgesia before skin incision. A midline vertical incision was made from the xiphoid to the 0.5-cm proximal pubic symphysis and explored the abdominal organs until the peritoneum for 1 min. Then, the abdominal incision was sutured layer by layer with 5-0 surgical sutures. At the end of the surgery, EMLA cream (2.5% lidocaine and 2.5% prilocaine) was applied to the incision site and then repeated every 8 h until 1 day postoperatively, to treat the pain associated with the incision. Several previous studies have shown that EMLA can meet the need for postoperative incisional analgesia in mice in the model of abdominal surgery (Zhang et al., 2013; Xu et al., 2014a,b). The surgical procedure was performed under sterile conditions and lasted around 10 min for each mouse, and the mouse was put back into the anesthesia chamber for the rest of the anesthesia up to 3 h. Finally, the mice were put back in the feeding cages after recovering from the anesthesia. The control group mice were stayed in their home cages with room air for 3 h, which is consistent with the condition of non-surgery patients. We used this method because surgery could enhance anesthesia neurotoxicity and such a combination of anesthesia and surgery has been shown to induce postoperative cognitive impairment and postoperative delirium-like behavior (Peng et al., 2016; Yang et al., 2017). And the model of surgery and anesthesia is more closely related to the clinical situation, which may provide theoretical reference for subsequent clinical studies.

### *N*-acetyl-cysteine and cyclosporin A application

*N*-acetyl-cysteine (Sigma-Aldrich) was given at 90 mg/kg in normal saline (adjust pH to 7.0 with NaOH) and the mice in the control plus NAC group and anesthesia/surgery plus NAC group were administered intraperitoneally (IP) at 0.5 h before the onset of sevoflurane exposure. CsA (MedChemExpress) was dissolved in 1% DMSO, and each mouse in the control plus CsA group and anesthesia/surgery plus CsA group was injected with CsA solution at a dose of 10 mg/kg through intraperitoneal (IP) at 0.5 h before the anesthesia/surgery. The NAC dose was based on studies using other models of oxidative stress with slight modification (Piao et al., 2020). The dose of CsA was based on studies of BBB integrity with slight modification (Bell et al., 2012).

### Behavior tests

**FIGURE 1:**
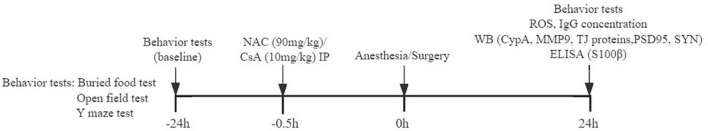
Timeline diagram of experimental procedures. Behavioral tests are performed 24 h before and 24 h after anesthesia/surgery. NAC (*N*-acetyl-cysteine) or CsA (Cyclosporin A) is injected intraperitoneally 0.5 h before anesthesia/surgery. ROS and IgG expressions in the prefrontal cortex; CypA, MMP9, TJ proteins, and PSD95, SYN levels in the prefrontal cortex; and S100β concentration in serum are detected.

Postoperative delirium is an acute neuropsychiatric disorder characterized by fluctuating disturbances in attention, level of consciousness, and cognition (Inouye et al., 2014). Consequently, we performed a variety of behavioral tests to identify natural behavior and cognitive function changes at 24 h before (baseline) and 24 h after the anesthesia/surgery. According to previously described methods, the behavior tests were carried out in the order of buried food test, open field test, and Y maze test (Peng et al., 2016), to simulate certain characteristics of clinical diagnosis in patients with POD. All mice were given 1 h to adapt to the environment before the behavior tests. And the field was wiped with alcohol and dried after each experiment to avoid the behavioral traces and the residual odor that interferes with the behavior of mice (Figure 1).

### Buried food test

To assess the natural tendency of mice to use olfactory cues for foraging, the buried food test was administered. Two days prior to the test, each mouse received two pieces of sweetened cereal every day at a fixed time. During the test, the test cage was prepared with 3 cm clean padding, and one piece of cereal was buried 0.5 cm below the padding (relatively fixed position). The latency was defined as the time from the mouse was placed in the test cage until the mouse held the food pellet with the front paws and started to eat, which was used to measure organized thinking and attention. The observation time was 5 min, and if the mouse failed to find the food within 5 min, the test was terminated, record the latency as 300 s. A new padding was replaced for each experiment.

### Open field test

Then, the mice were performed in the open field test to measure their exploratory and general activity. Under the dim light, each mouse was placed gently at the center of the open field box (50 × 50 × 50 cm) and allowed to move freely for 5 min. The following behavioral parameters were recorded by Any-Maze software (Stoelting Co., Wood Dale, IL, USA): total distance traveled, freezing time, latency to center, and time spent in the center.

### Y maze test

To further evaluate spatial learning and memory ability following anesthesia/surgery, the Y maze test was executed in a two-trial task. The Y maze has three arms, which were randomly set as the novel arm, the start arm, and the other arm, separated by a triangular central platform. The visual cues (in the form of colorful geometric shapes) were placed on the wall of each arm to help mice establish spatial memory. The Y maze test was divided into two trials separated by an inter-trial interval (ITI): the training trial and the test trial. During the training trial, the mouse was placed in the start arm to move 10 min freely in the start arm and the other arm, with the novel arm was blocked off. In the test trial, the novel arm was opened, and the mouse was placed in the start arm and allowed to explore freely all three arms for 5 min. For each mouse, the interval between the two phases was 2 h. And the percentage of novel arm entries and the time spent in the novel arm were measured.

### Brain tissue harvesting

At the end of behavior tests, mice were immediately anesthetized deeply with sevoflurane, and the blood was collected from the heart and then perfused with pre-cooled phosphate-buffered saline (PBS). The brain was removed and bisected in the mid-sagittal plane on ice, the left prefrontal cortex was harvested and stored at −80 °C for the western blot analysis, and the right cerebral cortex was used for fluorescence staining. The serum was analyzed for S100β using ELISA.

### Western blotting analysis

The prefrontal cortex tissue was added with RIPA lysate buffer (containing 1% PMSF and 1% phosphatase inhibitor) and then treated with ultrasound for 3 min on ice and centrifuged at 4 °C 12000 r/min for 10 min, and the supernatant was collected. The protein concentration was measured by bicinchoninic acid (BCA) protein assay kit. The proteins extracted from the prefrontal cortex were separated on SDS-polyacrylamide gels and transferred onto a polyvinylidene difluoride membrane, buffered and blocked with 5% skimmed milk powder at room temperature for 1 h. The membranes were incubated with the following primary antibodies overnight at 4 °C: rabbit polyclonal anti-ZO-1 antibody (1:1000, Invitrogen), rabbit monoclonal anti-PSD-95 antibody (1:1000, CST), rabbit monoclonal anti-MMP9 antibody (1:1000, Abcam), mouse monoclonal anti-Occludin antibody (1:800, Invitrogen), rabbit monoclonal anti-synaptophysin antibody (1:1000, CST), mouse monoclonal anti-Claudin5 antibody (1:1000, Invitrogen), mouse monoclonal anti-CypA antibody (1:200, Invitrogen), mouse monoclonal anti-β-actin antibody (1:1000, Promega). Then, the membranes were incubated with the secondary antibodies for 1 h at room temperature. Protein bands were visualized using chemiluminescence instruction (Amersham Imager 600). The protein band intensities of ZO-1, PSD-95, MMP9, Occludin, synaptophysin, Claudin5, and CypA were normalized to those of β-actin. The data of various experimental conditions were normalized to the results of their control animals.

### Immunofluorescence staining

The brain tissues were fixed in 4% paraformaldehyde at 4 °C overnight and immersed in 30% phosphate-buffered sucrose for 24 h, and then blocked in the Tissue-Tek OCT compound at −80 °C. Immunofluorescence was performed on 10 μm thick coronal sections. After being washed three times in PBS and permeabilized in 0.1% Triton X-100 for 10 min, sections were blocked in 2% fetal bovine serum (FBS) for 2 h at 37 °C and then incubated at 4 °C overnight with the following primary antibodies: mouse monoclonal anti-CypA antibody (1:50, Invitrogen) and rabbit monoclonal anti-PDGFRβ antibody (1:200, CST). Sections were rinsed in PBS and then incubated with the goat anti-mouse IgG antibody conjugated with Alexa Fluor 568 (1:200, Abcam), and goat anti-rabbit IgG antibody conjugated with Alexa Fluor 488 (1:200, Abcam) for 1 h at 37 °C in the darkness. Finally, immunostaining images were observed under a fluorescence microscope (Olympus BX53, Olympus, Japan).

### Quantitation of oxidative stress

Dihydroethidium (DHE, Beyotime) staining was used to detect ROS levels in the prefrontal cortex. Frozen sections from different groups were blocked using 5% bovine serum albumin (BSA) for 30 min at 37 °C, then incubated with PBS diluted dihydroethidium (DHE) at 37 °C for 30 min. The images were observed under a fluorescence microscope, in which the red fluorescence reflected the ROS content, and the fluorescent intensity was analyzed by Image J software.

### Evaluation of blood-brain barrier permeability

Immunoglobulin G (IgG) leakage was used to evaluate BBB permeability. In brief, the sections were fixed with 4% PFA for 10 min at room temperature, followed by staining with Cy-3-conjugated goat anti-mouse IgG (1:200, Affinity) for 2 h. After being mounted with a glass coverslip, the slides were scanned in a fluorescence microscope.

### Quantification of S100β

Serum samples were collected from heart blood in anesthetized mice, the levels of S100β in the serum of mice were measured following the manufacturer’s instructions of the ELISA kit (Cusabio, Wuhan, China).

### Statistical analysis

Quantitative data are expressed as mean ± standard error of the mean (SEM) unless otherwise stated. One-way analysis of variance (ANOVA) followed by Tukey’s *post-hoc* test was performed to determine differences in behavior tests between groups and differences in molecular biological data. GraphPad Prism version 7.0 for Windows (GraphPad Software, Inc., San Diego, CA, USA) was used to perform the statistical analysis. Differences with *p* < 0.05 were considered significant (*p < 0.05, ^**^p < 0.01, ^***^p < 0.001).

## Results

### Scavenging reactive oxygen species inhibits CypA/MMP9 pathway activation, attenuates blood-brain barrier disruption, and improves synaptic plasticity proteins in anesthesia/surgery mice

**FIGURE 2:**
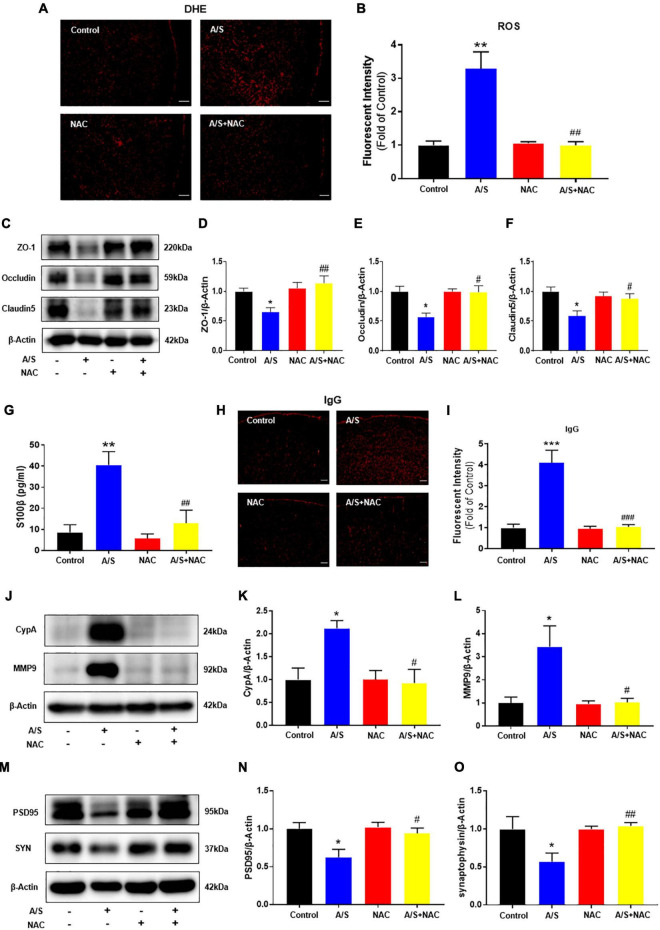
Effects of ROS on the CypA/MMP9 pathway, BBB permeability, and synaptic plasticity proteins in anesthesia/surgery mice. Scavenging of ROS improves the tight junction (TJ) proteins, attenuates the BBB leakage, and CypA/MMP9 activation, as well as increases synaptic plasticity proteins in anesthesia/surgery mice. The level of reactive oxygen species (ROS) in the prefrontal cortex was assessed by DHE staining **(A,B)**. Scale bars: 100 μm. The protein levels of CypA, MMP9 **(J–L)**, ZO-1, occludin, claudin5 **(C–F)**, PSD95, and synaptophysin **(M–O)** were assessed by western blotting. S100β levels in serum were measured by ELISA **(G)**. IgG leakage staining (red fluorescence) was shown in the cortex **(H,I)**. Scale bars: 100 μm. Values are expressed as mean ± SEM and were analyzed by one-way analysis of variance, followed by Tukey’s *post-hoc* test. *n* = 3–5 per group. A/S, anesthesia/surgery. *P < 0.05, **P < 0.01, ***P < 0.001, compared to control group; ^#^*P* < 0.05, ^##^*P* < 0.01, ^###^*P* < 0.001, compared to A/S group.

As a first step to determining the role of oxidative stress in anesthesia/surgery, the expression of ROS was measured by DHE staining. Compared with the control group, the fluorescence intensity of ROS was significantly increased in the anesthesia/surgery (A/S) group. NAC, a ROS scavenger, inhibited the increase of ROS post-anesthesia/surgery (Figures 2A,B). To find oxidative damage in the BBB components, changes in the expression of tight junction proteins occludin, claudin-5, and zonula occluden 1 (ZO-1) were examined, as they are considered to be sensitive indicators of structural changes in the BBB during disease pathogenesis (Zenaro et al., 2017). We found that anesthesia/surgery significantly decreased the levels of these TJ proteins, and that these changes were in line with previous studies (Yang et al., 2017) and significantly inhibited by NAC treatment (Figures 2C–F). Disruption of cerebral vascular barrier integrity as a result of TJ proteins injury was assessed by leaking in and leaking out of biomarkers across the BBB. The leaking out of brain matter into the blood circulation after anesthesia/surgery was analyzed by detecting S100β in blood samples. S100β is a calcium-binding protein mainly released by glial cells, and its release from the CNS into peripheral circulation is believed due to increased permeability of the BBB (Buttner et al., 1997). In this study, mice in the anesthesia/surgery group showed a significantly higher level of S100β in the blood samples compared with controls, while NAC reversed these changes (Figure 2G). Under the condition of BBB breakdown, IgG, along with other plasma components, can escape from blood vessels and enter the brain interstitial space (Abbott et al., 2010; Acharya et al., 2015). Therefore, we used IgG immunofluorescence staining to probe for the presence of interstitial IgG as a visual indicator of vascular leakage and BBB breakdown. We observed that compared with the control group, the fluorescence intensity of IgG significantly increased after anesthesia/surgery, which was ameliorated by NAC treatment (Figures 2H,I). These results indicate that ROS play a critical role in the BBB damage induced by anesthesia/surgery. We next examined the possible mechanisms of BBB damage challenged by ROS. Given that cells can secrete CypA in response to oxidative stress (Suzuki et al., 2006), and the CypA/MMP9 pathway has been shown to mediate BBB damage (Bell et al., 2012). Therefore, we investigated whether anesthesia/surgery increase the expression of CypA/MMP9, and the effect of NAC on CypA/MMP9 levels after anesthesia/surgery was evaluated. We observed that anesthesia/surgery induced a significant increase of CypA/MMP9 in the prefrontal cortex, while NAC treatment reversed this effect (Figures 2J–L). Synaptic proteins including postsynaptic density protein (PSD)-95 and synaptophysin (SYN), with the reductions of these synaptic markers suggesting synaptic loss, potentially leading to cognitive impairment (Hong et al., 2016). Compared with the control group, the levels of PSD95 and SYN in the prefrontal cortex were markedly reduced after anesthesia/surgery. However, NAC treatment attenuated these changes (Figures 2M–O). These results indicated that intraperitoneal administration of NAC significantly attenuated oxidative stress, CypA/MMP9 activation, BBB disruption, and synaptic plasticity proteins disorder following anesthesia/surgery, as convinced by preventing the disruption of TJ proteins ZO-1, occludin, and claudin-5, followed by the decrease of ROS.

### Scavenging reactive oxygen species attenuates delirium-like behavior in anesthesia/surgery mice

**FIGURE 3:**
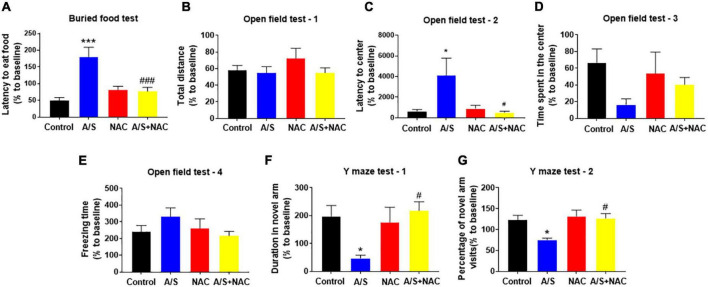
Effects of ROS on anesthesia/surgery-induced delirium-like behavior in mice. Scavenging ROS alleviates anesthesia/surgery-induced delirium-like behavior. In the buried food test, CsA decreased the latency to eat food **(A)** in mice. In the open field test anesthesia/surgery had no influence on total distance **(B)**, time spent in the center **(D)** or freezing time **(E)**. CsA decreased the latency to center **(C)** in mice. In the Y maze test, CsA increased the duration in novel arm **(F)** and the percentage of novel arm visits **(G)** in mice with delirium-like behavior. Values are expressed as mean ± SEM and were analyzed by one-way analysis of variance, followed by Tukey’s *post-hoc* test. *n* = 10 per group. A/S, anesthesia/surgery. *P < 0.05, ***P < 0.001, compared to control group; ^#^*P* < 0.05, ^###^*P* < 0.001, compared to A/S group.

Given the findings that NAC attenuated the anesthesia/surgery-induced BBB breakdown and synaptic plasticity proteins deficits, we next determined whether NAC could also ameliorate the anesthesia/surgery-induced behavior changes. The buried food test was used to evaluate the natural behavior of aged mice, as the ability to find and eat the food depends on complete attention, organized thinking, and a normal level of consciousness (Peng et al., 2016). The mice in the A/S group displayed longer latency to find the hidden food within 5 min than those in the control group, which was reversed by NAC treatment (Figure 3A). The open field was used to analyze locomotion and anxiety-like behavior in rodents (Kraeuter et al., 2019b), and locomotor activity was quantified based on total distance traveled, with the time spent in the central area and the latency to first enter the center reflecting the anxiety level. During the 5-min test session, there were no significant differences in the total distance, and the freezing time, or the time spent in the center of the arena among the four groups (Figures 3B,D,E). However, the latency to the center area was significantly longer in the A/S group than those in the control group, which was attenuated by NAC (Figure 3C). We employed the Y maze test to assess the changes in spatial learning and memory ability in mice (Kraeuter et al., 2019a). And compared with the control group, the time spent in the novel arm and the percentage of novel arm entries were significantly decreased in the A/S group. This reduction was not observed in the anesthesia/surgery plus NAC groups (Figures 3F,G). These results suggested that anesthesia and surgery induce the dysfunction of natural and learning behaviors in aged mice and that NAC attenuated this dysfunction, indicating the role of ROS in the effects.

### Inhibition of CypA/MMP9 pathway improves blood-brain barrier disruption and synaptic plasticity proteins decrease

**FIGURE 4:**
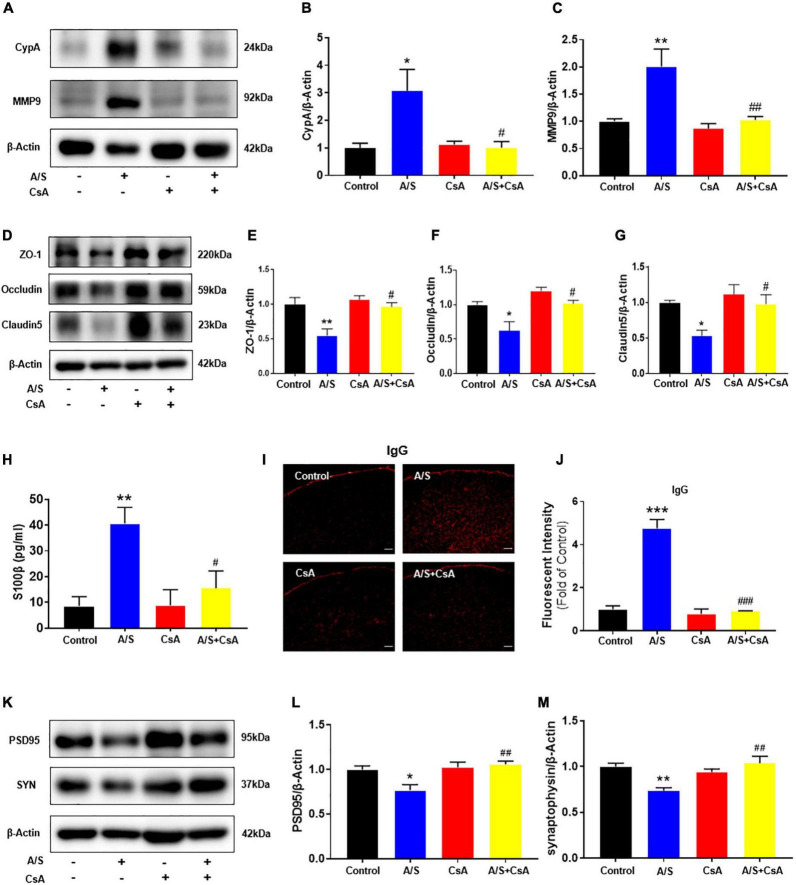
Effects of CypA/MMP9 pathway on BBB permeability and synaptic plasticity proteins in anesthesia/surgery mice. Inhibition of CypA/MMP9 pathway improves the tight junction (TJ) proteins, attenuates the BBB leakage, and increases synaptic plasticity proteins in anesthesia/surgery mice. The protein levels of CypA, MMP9 **(A–C)**, ZO-1, occludin, claudin5 **(D–G)**, PSD95 and synaptophysin **(K–M)** were assessed by western blotting. S100β levels in serum were measured by ELISA **(H)**. IgG leakage staining (red fluorescence) was shown in the cortex **(I,J)**. Scale bars: 100 μm. Values are expressed as mean ± SEM and were analyzed by one-way analysis of variance, followed by Tukey’s *post-hoc* test. *n* = 3–5 per group. A/S, anesthesia/surgery. *P < 0.05, **P < 0.01, ***P < 0.001, compared to control group; ^#^*P* < 0.05, ^##^*P* < 0.01, ^###^*P* < 0.001, compared to A/S group.

Since the CypA/MMP9 pathway has been confirmed to participate in regulating the structure and permeability of the BBB (Montagne et al., 2020), the activation of MMPs by oxidative stress is involved in the digestion of the tight junction and basement membrane proteins (Pun et al., 2009). We focused our investigation on the role of CypA/MMP9 mediated by ROS on the degradation of BBB. Immunofluorescent staining showed that CypA was co-localized with the staining of PDGFRβ, a pericyte marker (Supplementary Figure 2), suggesting that CypA is mainly expressed in the pericytes after anesthesia/surgery. As shown above, the ROS scavenger NAC attenuated the anesthesia/surgery-induced release of CypA/MMP9. To further demonstrate the role of CypA/MMP9 pathway in BBB permeability, we used CsA, an inhibitor of CypA, which significantly decreased the expression of CypA/MMP9 (Figures 4A–C), and then increased ZO-1, occludin, and claudin5 levels (Figures 4D–G) compared to the A/S group. Meanwhile, CsA significantly reduced the fluorescence intensity of IgG in the cortex and inhibited the release of S100β in the serum after anesthesia/surgery (Figures 4H–J). Furthermore, CsA treatment rescued the reduction of PSD95 and SYN levels caused by anesthesia/surgery (Figures 4K–M). These results suggest that anesthesia/surgery caused BBB breakdown possibly *via* the ROS-mediated CypA/MMP9 signaling pathway.

### Inhibition of CypA/MMP9 pathway attenuates delirium-like behavior in anesthesia/surgery mice

**FIGURE 5:**
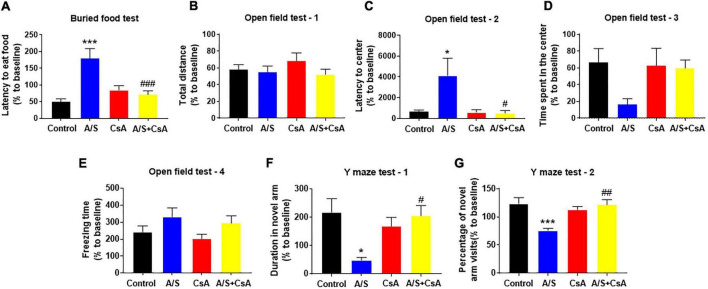
Effects of CypA/MMP9 pathway on anesthesia/surgery-induced delirium-like behavior in mice. Inhibition of CypA/MMP9 pathway improves anesthesia/surgery-induced delirium-like behavior. In the buried food test, CsA decreased the latency to eat food in mice **(A)**. In the open field test, anesthesia/surgery had no influence on total distance **(B)**, time spent in the center **(D)** or freezing time **(E)**. CsA decreased the latency to center **(C)** in mice. In the Y maze test, CsA increased the duration in novel arm **(F)** and the percentage of novel arm visits **(G)** in mice with delirium-like behavior, which is induced by anesthesia/surgery. Data are expressed as mean ± SEM and were analyzed by one-way analysis of variance, followed by Tukey’s *post-hoc* test. The control and A/S groups are shared between Figure 3. *n* = 10 per group. A/S, anesthesia/surgery. *P < 0.05, ***P < 0.001, compared to control group; ^#^*P* < 0.05, ^##^*P* < 0.01, ^###^*P* < 0.001, compared to A/S group.

Due to the inhibition of CypA/MMP9 could attenuate anesthesia/surgery-induced BBB damage and synaptic plasticity proteins decrease, we next examined whether CsA could reverse delirium-like behavior induced by anesthesia/surgery. In the buried food test, CsA pretreatment significantly decreased the latency to eat food compared to mice in the A/S group (Figure 5A). Additionally, CsA pretreatment significantly decreased the latency to the center compared to the A/S group in the open field test (Figure 5C), with the situation that there were no significant differences in total distance traveled, the freezing time, or the time spent in the center at 24 h after anesthesia/surgery among mice in the four groups (Figures 5B,D,E). Moreover, in the Y maze test, CsA pretreatment significantly increased the percentage of entries and prolonged the duration in the novel arm compared to the A/S group (Figures 5F,G). In general, these findings demonstrated that the CypA/MMP9 signaling pathway was the potential mechanism for delirium-like behavior.

## Discussion

Anesthesia and surgery have been reported to induce delirium-like behavior in rodents (Peng et al., 2016; Zhang et al., 2018; Liu et al., 2022), in which oxidative stress and BBB dysfunction play important roles (Karlidag et al., 2006; Maldonado, 2013; Yang et al., 2017; Zhang et al., 2018; Taylor et al., 2022). To explore the potential mechanisms by which ROS induce delirium-like behavior, this study mainly addressed the following research questions: First, does ROS mediate delirium-like behavior by disrupting BBB integrity? Second, how does ROS regulate BBB disruption? A battery of behavior tests was measured to evaluate the behavior changes after anesthesia/surgery. We studied BBB integrity by examining the levels of TJ proteins and the extravasation of IgG and S100β. Our results showed that ROS mediated the harmful effects on BBB integrity and delirium-like behavior after anesthesia/surgery, potentially *via* the CypA/MMP9 signaling pathway. These findings may indicate a new approach for preventing POD, while CypA is a potential target. To the best of our knowledge, this has not been reported before.

Reactive oxygen species are highly reactive chemical molecules produced by the imbalance between oxidants and antioxidants in the body, and play a pivotal role in the pathogenesis mechanism of neurodegeneration (Sayre et al., 2001; Patel, 2016). ROS produced excessively would mediate detrimental effects, which are known as oxidative stress (Rosenfeldt et al., 2013). It has been demonstrated that perioperative oxidative stress is associated with the increased risk of postoperative delirium (Karlidag et al., 2006; Lopez et al., 2017, 2020; Kazmierski et al., 2021). Similarly, in the present study, we found that anesthesia/surgery induced ROS accumulation and delirium-like behavior, while the ROS scavenger NAC attenuated these changes. Cognitive decline is a common feature of aging with complex mechanisms, and some scholars have shown that cerebral microvascular dysfunction is one of the important factors (Toth et al., 2017). Patients with AD and small vessel disease have observed changes in cerebral microvascular density and morphology, loss of tight junctions, and leakage of BBB (Tarantini et al., 2017). Inflammatory mediators and neurovascular dysfunction-induced BBB breakdown was associated with POD (Taylor et al., 2022), possibly toward subsequent dementia (Davis et al., 2012). Further, BBB dysfunction can potentially exacerbate neuroinflammation, resulting in neurodegenerative pathology, and serve as a key process in the development of neuroinflammation (Takata et al., 2021). Studies are underway to evaluate BBB dysfunction as a risk factor for postoperative delirium. Several animal models have reported that anesthesia/surgery can decrease the levels of TJ proteins, and increase the leakage of BBB or blood-cerebrospinal barrier (BCB) accompanied by delirium-like behavior (Yang et al., 2017; Wen et al., 2020; Chen et al., 2021; Li et al., 2021). The present study reconfirmed that BBB disruption was associated with anesthesia/surgery-induced delirium-like behavior. This disruption has also been found in several neurological disorders, such as traumatic injury, stroke, and neurodegenerative diseases (Persidsky et al., 2006). Thus, understanding the pathogenesis of anesthesia/surgery-mediated BBB destruction may ultimately lead to effective prevention of postoperative memory and attention deficits.

As mentioned in a related review, ROS have been strongly implicated in providing a common trigger for many downstream pathways that mediate BBB compromise (Pun et al., 2009). Zehendner et al. (2013) reported that ROS mediate a crucial role in regulating BBB permeability under the situation of moderate hypoxia followed by reoxygenation, likely by regulating TJ proteins and brain endothelial cell (BEC) integrity. In traumatic brain injury, oxidative stress induced cerebral vascular BBB leakage *via* activating MMPs (Abdul-Muneer et al., 2013, 2015). Also, under the condition of hypoxic, melatonin proved to be useful in ameliorating the NO-mediated increase in BBB permeability (Kaur and Ling, 2008). Our results were in accord with related studies and showed that the ROS scavenger NAC significantly restored BBB permeability and alleviated delirium-like behavior induced by anesthesia/surgery. The role of anesthesia/surgery in increasing BBB permeability and the capacity of NAC to strengthen the BBB suggested that ROS-mediated BBB breakdown can contribute, at least partially, to the underlying mechanism of the anesthesia/surgery-induced delirium. Future studies would include the determination of other antioxidants, such as melatonin or Vitamin E, that could be used to prevent or treat POD. Cerebral microvascular alterations can directly affect neuronal and synaptic functions through changes in the blood flow and the BBB permeability (Zlokovic, 2008). BBB breakdown, due to the destruction of the Tjs, leads to progressive synaptic and neuronal dysfunction (Zlokovic, 2008). Similarly, we found that synaptic plasticity proteins were reduced after anesthesia/surgery, while treatment with NAC or CsA rescued these changes and accompanied the recovery of BBB damage. NAC is a high safety profile agent (Skvarc et al., 2017) that reduces oxidative stress and inflammation (Skvarc et al., 2016) and exerts beneficial effects for the treatment of cognitive decline in animal models (Bavarsad Shahripour et al., 2014). There are many clinical studies demonstrating that NAC may be beneficial in the prevention of cognitive decline associated with psychiatric and dementia-related disorders (Smaga et al., 2021), including bipolar disorder, post-traumatic stress disorder, late-stage Alzheimer’s disease (Adair et al., 2001), and others. Based on the fact that other antioxidants like α-tocopherol and selegiline have shown positive effects on the progression of cognitive decline in clinical studies (Sano et al., 1997). NAC may be a safe and effective agent in the future clinical treatment of POD.

Next, we explored the mechanism by which ROS regulate BBB permeability in mice with delirium-like behavior. CypA is regarded as a specifical leukocyte chemotactic factor that regulates a variety of cellular signaling pathways (Suzuki et al., 2006) and the activation of CypA can increase the secretion of MMP9 (Pan et al., 2020), a well-known downstream effector that degrades TJ proteins (Turner and Sharp, 2016). Accumulating evidences have suggested that the CypA/MMP9 pathway is involved in regulating the structure and function of BBB (Halliday et al., 2016; Montagne et al., 2020). Nevertheless, the effect of the CypA/MMP9 pathway on ROS-regulated BBB permeability remains largely to be determined. Previous studies have shown that CypA is mainly expressed in the pericytes in the brain (Halliday et al., 2016; Pan et al., 2020). Consistent with this finding, CypA was mostly expressed in pericytes following anesthesia/surgery. In models of subarachnoid hemorrhage (SAH) (Pan et al., 2017), ischemic stroke (Deng et al., 2020), and traumatic brain injury (Main et al., 2018), CsA has been shown to have a protective effect on the BBB by inhibiting CypA/MMP9 pathway and improving neurocognitive function, which was also noted in our model. We found that pretreatment with the CypA inhibitor CsA alleviated BBB damage and delirium-like behavior induced by ROS production, indicating that ROS may regulate BBB permeability and delirium-like behavior *via* the CypA/MMP9 pathway, in which CypA is a potential therapeutic target for preventing POD. CsA could reverse the development of delirium-like behavior by inhibiting CypA, and that short-term administration with low-dose CsA at 10 mg/kg may be a promising treatment for postoperative cognitive decline in mice (Peng et al., 2016; Zhang et al., 2018). However, some *in vitro* cellular experiments have shown a dose-dependent effect of CsA on increased BBB permeability (Dohgu et al., 2004, 2010), which is related to the inhibition of P-gp activity on the BBB (Dohgu et al., 2007). Meanwhile, the immunosuppressive side effects (Liddicoat and Lavelle, 2019) and dose-dependent toxicity (Patocka et al., 2021) of CsA may be confounding factors in this study, which may restrict its potential clinical application in preventing and treating the development of POD.

The innovative discovery of this study was that the upstream and downstream relationships between ROS and CypA has been basically verified. Since cells can secrete CypA in reaction to oxidative stress (Suzuki et al., 2006), it is conceivable that ROS may be the upstream molecule that activates CypA-mediated BBB destruction. Our results showed that the ROS scavenger NAC reversed the activity of CypA/MMP9 and protected BBB from damage. These above results revealed important mechanisms that anesthesia/surgery increase CypA expression and offer new insights into the role of ROS in the aberrant activation of CypA-mediated MMP9 signaling. These findings also provide an opportunity to design new interventions to prevent or treat possible anesthesia/surgery-induced POD in elderly people. CsA has been reported to suppress oxidative stress by inhibiting the mitochondrial permeability transition pore (mPTP) opening (Antoniel et al., 2014). Nevertheless, our results showed that pretreatment with CsA had no significant effect on the levels of ROS after anesthesia/surgery in aged mice (Supplementary Figure 1). Zhang et al. (2018) reported that CsA attenuated the decreased levels of anti-oxidative stress biomarkers and inhibited the increased ROS levels after anesthesia/surgery in 8-week-old mice. Another interesting study indicated that CsA selectively attenuated the anesthesia/surgery-induced reduction in adenosine triphosphate (ATP) levels without affecting the ROS accumulation in 4-month-old mice (Peng et al., 2016). We speculate that the difference may depend on different main sources and degrees of oxidative stress between young and old mice after anesthesia/surgery.

However, there are several limitations in the present study. First, our results showed that NAC and CsA can alleviate BBB damage by scavenging ROS or inhibiting CypA pathway, suggesting that NAC and CsA may have similar effects on preventing the development of delirium-like behavior, but their interactions were not clear. Therefore, we did not measure a co-treatment group. In the next study, we will set up a combination treatment group and further investigate which drug plays the major role in preventing the development of POD. Second, this study mainly explored the neuropathogenesis of POD in the PFC, as the PFC plays a key role in executive function and cognitive control (McEwen and Morrison, 2013). Recent imaging studies have revealed that in the active phase of delirium, the function between different regions of the cortex was mainly destroyed, and in the subcortical regions the functional connectivity was reversibly reduced but showed no abnormalities in the functional connectivity of the hippocampus (Choi et al., 2012; Oh and Park, 2019). Thus, delirium is defined as an acute brain dysfunction and inattention is its prominent feature, which points to the importance of the PFC (Culley et al., 2014). Third, our data showed that ROS are molecules upstream of CypA that mediate anesthesia/surgery-induced BBB damage and delirium-like behavior. In light of the apparently important role of ROS in regulating BBB integrity in POD mice following anesthesia/surgery. Future studies will include determining the possible relationship between the major sources of oxidative stress, changes in the expression and/or activities of pro-oxidant and antioxidant enzymes, and the alteration of neuroinflammation in association with BBB disruption. To provide insights for clinical studies to assess perioperative indicators of oxidative stress and neurovascular integrity and their potential link to perioperative neurocognitive impairment, as well as to guide effective preventive or therapeutic interventions.

**FIGURE 6:**
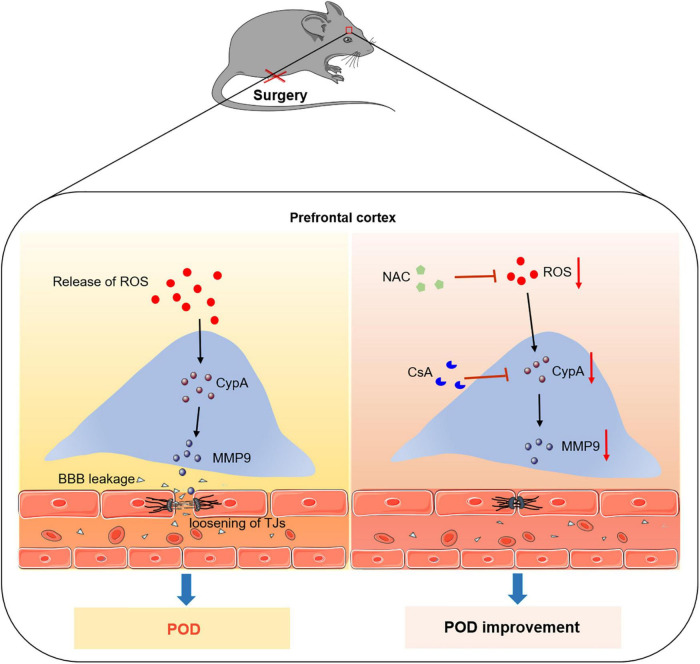
Proposed pathway in the study. We find that the ROS scavenger alleviates anesthesia/surgery-induced CypA/MMP9 activation, BBB damage, and delirium-like behavior in mice. Furthermore, CypA inhibitor prevents BBB damage and delirium-like behavior by suppressing CypA/MMP9 pathway. These findings suggest that the ROS mediated CypA/MMP-9 pathway is a mechanism for inducing BBB injury and delirium-like behavior after anesthesia/surgery in aged mice.

In conclusion, our results provide a new mechanism for the development of POD. Anesthesia/surgery induce the release of ROS, and then activate the expression of CypA/MMP9 cascade, resulting in BBB dysfunction and delirium-like behavior. Thus, CypA may be a potential molecular target for preventing POD (Figure 6).

## Data availability statement

The datasets presented in this study can be found in online repositories. The names of the repository/repositories and accession number(s) can be found in the article/Supplementary material.

## Ethics statement

The animal study was reviewed and approved by the Ethical Regulation on the Care and Use of Laboratory Animals of Anhui Medical University.

## Author contributions

G-HX designed the study. L-FL wrote the manuscript. L-FL, YH, Y-NL, D-WS, CL, XD, and S-HZ performed the research and analyzed the data. Q-YZ, J-QZ, and G-HX revised the manuscript. All authors contributed to the article and approved the submitted version.
