# Supplementary material for: Reactive oxygen species contribute to delirium-like behavior by activating CypA/MMP9 signaling and inducing blood-brain barrier impairment in aged mice following anesthesia and surgery
Source: Front Aging Neurosci. 2022 Oct 19;14:1021129. doi: 10.3389/fnagi.2022.1021129 (PMC9629746; doi:10.3389/fnagi.2022.1021129)
Supplement: Supplementary file 1 [file Data_Sheet_1.pdf]

Supplementary Material

Supplementary Figure 1

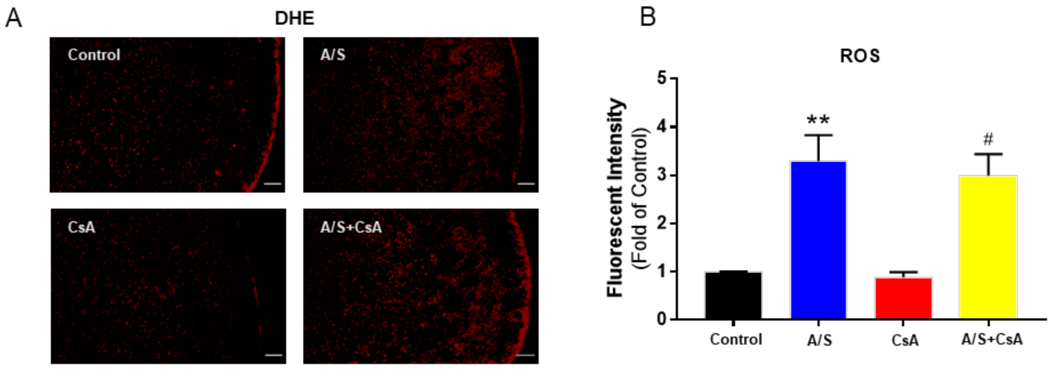

Figure 1. Effect of CsA on the anesthesia/surgery-induced expression of ROS. CsA has no significant inhibitory effect on anesthesia/surgery-induced ROS expression. The level of reactive oxygen species (ROS) in the prefrontal cortex was assessed by DHE staining (A), and quantitative results were shown (B). Scale bars: 100 μm. Values are expressed as mean ± SEM and were analyzed by one-way analysis of variance, followed by Tukey's post-hoc test. n = 3 per group. A/S: anesthesia/surgery. ** $P < 0.01$, compared to control group; $^{\#}$ $P < 0.05$, compared to CsA group.

Supplementary Figure 2

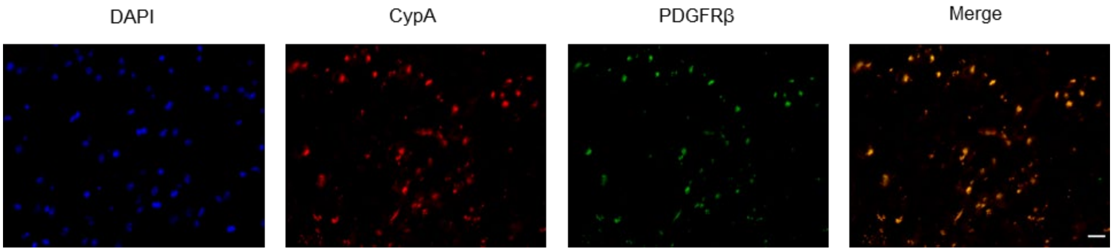

Figure 2. Immunofluorescence staining of CypA and pericytes marker. Fluorescent in situ hybridization to analyze the source of CypA post-anesthesia/surgery. CypA alongside the marker of pericytes and the majority expression of CypA was in PDGFRβ-positive pericytes. Scale bar: 20 μm.
